# Recycling lead and transparent conductors from perovskite solar modules

**DOI:** 10.1038/s41467-021-26121-1

**Published:** 2021-10-06

**Authors:** Bo Chen, Chengbin Fei, Shangshang Chen, Hangyu Gu, Xun Xiao, Jinsong Huang

**Affiliations:** Department of Applied Physical Sciences, University of North Carolina at Chapel Hill, Chapel Hill, NC 27599 USA

**Keywords:** Environmental impact, Solar energy

## Abstract

Perovskite photovoltaics are gaining increasing common ground to partner with or compete with silicon photovoltaics to reduce cost of solar energy. However, a cost-effective waste management for toxic lead (Pb), which might determine the fate of this technology, has not been developed yet. Here, we report an end-of-life material management for perovskite solar modules to recycle toxic lead and valuable transparent conductors to protect the environment and create dramatic economic benefits from recycled materials. Lead is separated from decommissioned modules by weakly acidic cation exchange resin, which could be released as soluble Pb(NO_3_)_2_ followed by precipitation as PbI_2_ for reuse, with a recycling efficiency of 99.2%. Thermal delamination disassembles the encapsulated modules with intact transparent conductors and cover glasses. The refabricated devices based on recycled lead iodide and recycled transparent conductors show comparable performance as devices based on fresh raw materials. Cost analysis shows this recycling technology is economically attractive.

## Abstract (web summary)

Perovskite photovoltaics has become more competitive against silicon counterpart in reducing cost of solar energy, yet the management of toxic lead hampers it application. Here, the authors propose a cost-effective environmental-friendly approach to recycle lead and transparent conductors.

## Introduction

Perovskite photovoltaic (PV) technologies are revolutionizing electricity generation by using a new generation of metal halide perovskites (MHPs)^1,2^. The best perovskite solar-cell efficiency already reached 25.5%, comparable to the best PV cells made of single-crystal silicon, and the perovskite/silicon tandem solar cells already achieved a high certified efficiency of 29.5%^3^. The defect-tolerant metal halide perovskites could be manufactured by low-cost solution processes, such as blade coating, slot-die coating, and spray coating, which offer small capital expense^4–8^. The efficiencies of perovskite minimodules fabricated by scalable deposition methods are also approaching 20%^9^. There are more than a dozen companies world-widely commercializing the perovskite PV either by combining with existing PV technology using tandem structures, or standalone using single-junction structure^10^.

Most efforts in industry and academia have been focusing on upscaling and enhancing module/cell efficiency and stability for perovskite PV. However, most efficient metal halide perovskites for this purpose contain toxic lead, such as α-FAPbI_3_^11,12^, (FAPbI_3_)_0.95_(MAPbBr_3_)_0.05_^13^, MA_x_FA_1−x_PbI_3_^14,15^, etc. Although there have been extensive attempts to replace lead in MHPs, all current lead-free perovskite solar cells suffer from either much poorer stability, such as the tin-based perovskite solar cells^16,17^, or much lower efficiency, such as double-perovskite-based solar cells^18^, compared with their lead-based counterparts. One gigawatt of solar PV capacity using perovskite solar panels with efficiency of 20% would contain ~3.5 tons of lead using the best-known perovskite materials as listed above, assuming a perovskite film thickness of 500 nm. The perovskite solar panels will contain up to ~6000 tons of lead, if only 20% of anticipated 8500-gigawatt PV market in 2050 is occupied by perovskite PV^19^. Recently, lead-adsorbing materials, such as P,P′-di(2-ethylhexyl)methanediphosphonic acid and sulfonic acid cation-exchange resin, have been reported to integrate in perovskite solar panels to prevent lead leakage from damaged perovskite solar modules^20–22^. Besides this lead management during operation in field, a lead management for end-of-life perovskite solar modules now needs top priority to ensure the future of these technologies, otherwise, the promised low-cost advantages of perovskite solar cells cannot be realized.

It is still a challenge to properly dispose and recycle the silicon solar panels at the end-of-life, which becomes an urgent task due to the solar panels installed in 2000s are reaching the end of their lifespan^23–25^. Even though the silicon solar panels contain many valuable materials (such as silicon, glass, silver, aluminum, etc.), it still lacks a cost-effective recycling technology to recover those materials, and thus most decommissioned silicon panels still go to landfill^23–25^. When it comes to perovskite solar modules that contain toxic and water soluble lead, going to landfill is not an option due to its threat to ecosystem and human health. It is essential to develop a practical recycling technique, especially lead-recycling technique, for perovskite solar modules^26–30^. Several lead-removal methods have been reported for wastewater treatment, such as chemical precipitation, electrodeposition, ion exchange, membrane separation, and adsorption^31–36^. However, they are established for aqueous pollutants, while the perovskite solar modules require organic solvents for high lead solubility and recycling capacity, where a cost-effective technique has not been developed yet. Recently, Park et al. reported iron-incorporated hydroxyapatite as a new adsorbent to recover lead from perovskite-containing organic solvent^37^. But it lacks cost analysis for lead recycling through the complicated iron-incorporated hydroxyapatite hollow composites, and this adsorbent cannot be reused due to its dissolving during lead-release process^37^.

In this study, we proposed a low-cost recycling technique for perovskite solar modules using carboxylic acid cation-exchange resin as lead adsorbent and using thermal delamination to expose perovskite films. The carboxylic acid cation-exchange resin could efficiently adsorb lead ions from organic solvents and efficiently release the adsorbed lead ions to clean solution by ion-exchange process between Pb^2+^ ions and H^+^ ions. Different from previous lead-trapping studies via strongly acidic cation-exchange resin^21,22^, the weakly acidic cation-exchange resin showed better lead recycling efficiency due to easy release of Pb^2+^ ions from carboxylic acid functional group compared with sulfonic acid. The lead was precipitated from aqueous solution as recrystallized PbI_2_ for reusing after reaction with sodium iodide. We also developed a thermal delamination method to disassemble the encapsulated perovskite solar module with intact glass substrates, which recycled both front transparent conductor and back cover glass with high reuse value. Finally, the recycling cost was estimated to evaluate the economic incentives of this recycling technique.

## Results

### Recycling roadmap

**Fig. 1:**
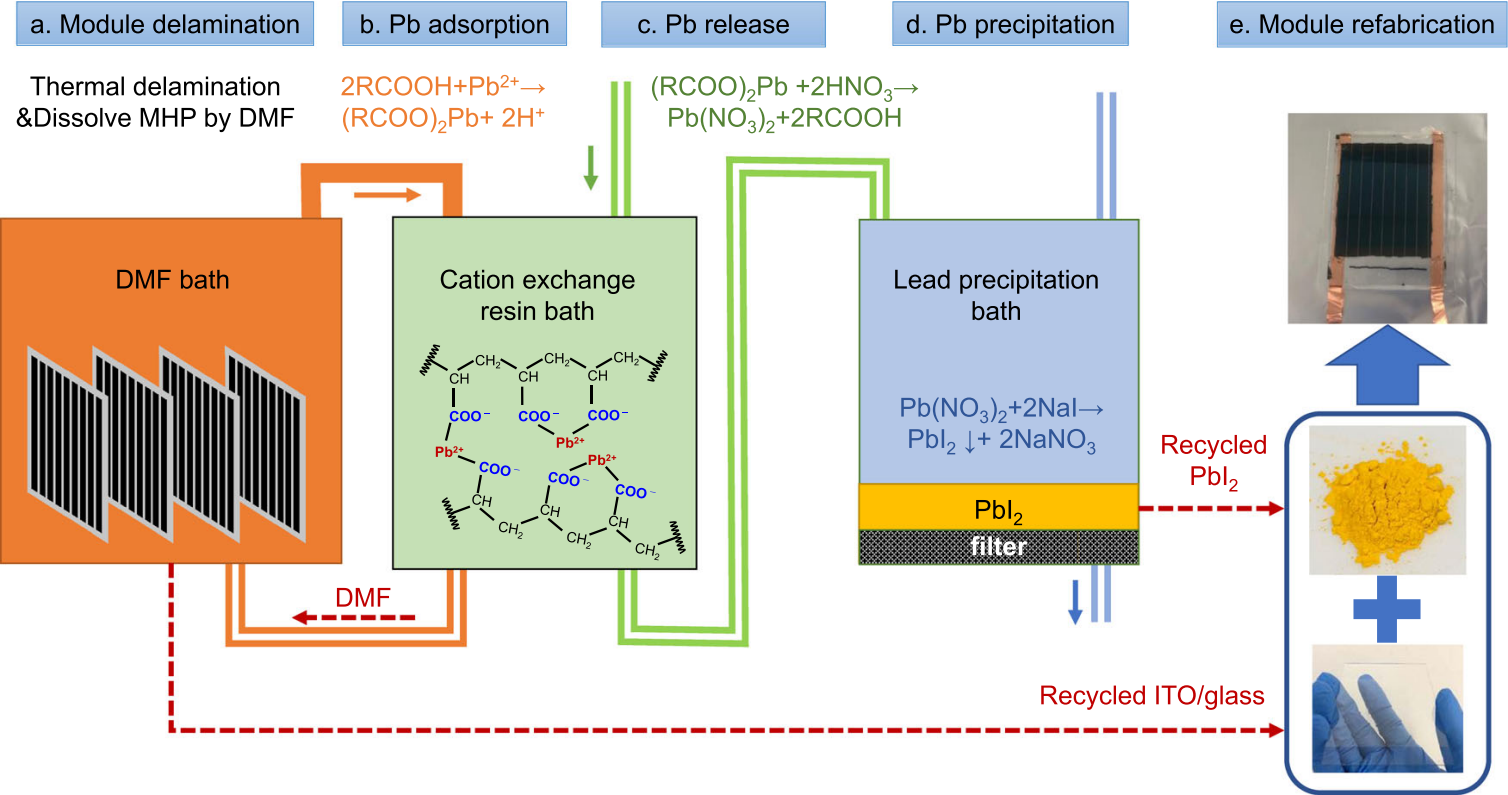
Roadmap for recycling of perovskite solar modules. **a** Encapsulated perovskite solar modules were delaminated and MHP was dissolved by DMF. **b** Lead ions in DMF were removed by carboxylic acid cation-exchange resin. **c** The adsorbed lead ions on resin were released to aqueous solution by resin-regeneration process via HNO_3_. **d** Precipitation of PbI_2_ by pouring NaI into Pb(NO_3_)_2_ containing solution. **e** Module refabrication based on recycled materials.

The proposed roadmap to recycle toxic lead and valuable glass substrates from perovskite solar modules is sketched in Fig. 1. After the delamination of encapsulated perovskite solar modules, the lead in perovskite layer is dissolved by organic solvent, such as dimethylformamide (DMF). Lead ions are first adsorbed by a lead adsorbent to fully remove lead in the organic solvent, and then are released to clean solvent, followed by a precipitation to PbI_2_ for reuse. In this study, we chose carboxylic acid cation-exchange resin as adsorbent to recycle lead in decommissioned perovskite solar modules. The lead-adsorption process and lead-release process on resins are based on ion exchange between H^+^ ions and Pb^2+^ ions:

1 $$2{{{{{\rm{R}}}}}}-{{{{{\rm{COOH}}}}}}+{{{{{{\rm{Pb}}}}}}}^{2+}\leftrightarrow {\left({{{{{\rm{R}}}}}}-{{{{{\rm{COO}}}}}}\right)}_{2}{{{{{\rm{Pb}}}}}}+{2{{{{{\rm{H}}}}}}}^{+}$$

It is noted that the ion-exchange process is a reversible reaction. A high concentration of H^+^ ions in solution could reverse the equilibrium in equation (1), which is a resin-regeneration process and thus can be used for lead release. Typical regenerant for cation-exchange resins is high-concentration HCl or H_2_SO_4_ acid solution. Considering PbCl_2_ and PbSO_4_ have low solubility in aqueous solution, regeneration by HCl and H_2_SO_4_ could directly precipitate the released Pb^2+^ ions as PbCl_2_ and PbSO_4_, respectively, which cause difficulty to separate lead with resin. Here, we chose HNO_3_ aqueous solution as regenerant to release the adsorbed lead ions as water-soluble Pb(NO_3_)_2_. Because PbI_2_ is the main lead source for most of highly efficient perovskite solar cells, the best form of recycled lead for reusing is PbI_2_ Therefore, the lead is converted from Pb(NO_3_)_2_ to PbI_2_ precipitation by adding low-cost NaI due to their solubility difference in aqueous solution.

### Module delamination

**Fig. 2:**
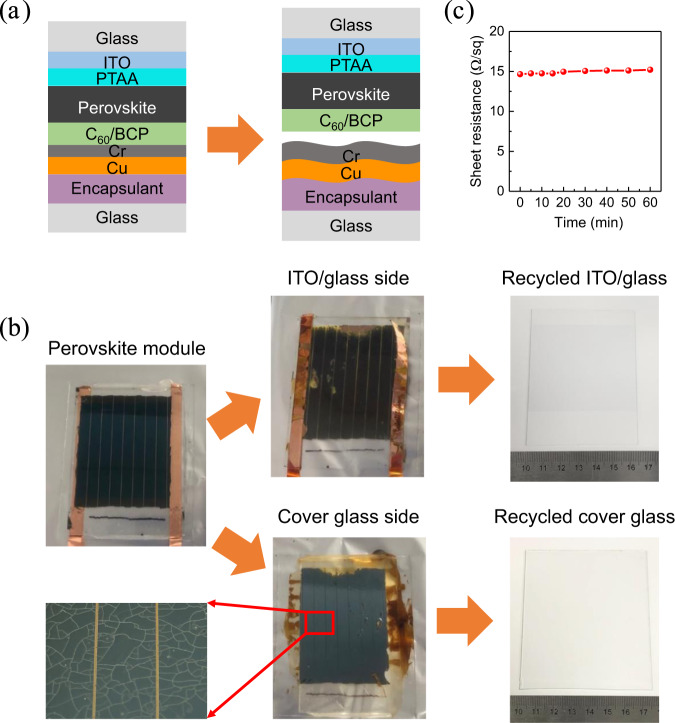
Thermal delamination of encapsulated perovskite solar module. **a** Schematic illustration for delamination at the interface of electron-transport layer and metal electrode. **b** Photo of an encapsulated perovskite solar module, delaminated module at ITO/glass side and back cover-glass side, and recycled ITO/glass and cover glass after cleaning. The front glass size is 8.5 cm × 6.5 cm. **c** Change of sheet resistance for ITO/glass substrate after thermal annealing at 250 °C for 1 h under ambient atmosphere.

To recycle the end-of-life perovskite solar modules, the first step is to develop a delamination technique to disassemble the encapsulated modules in order to expose the perovskite layer. Here we consider the perovskite module structure fabricated on indium tin oxide (ITO) glass and encapsulated with another piece of glass and encapsulant. The existing encapsulants for perovskite solar cells include epoxy resin, polyolefin, Surlyn, polyisobutylene, polyurethane, etc., together with a back cover glass which can effectively prevent the permeation of moisture, oxygen, and other hazards^38–41^. This is the most likely structure in commercial perovskite solar modules that gives best stability, and was thus chosen for this study. We discovered that a short thermal treatment at high temperature can effectively disassemble the encapsulated perovskite solar modules and obtain both intact ITO glass and back cover glass. After thermal stress at 250 °C for 2 min, the polymer encapsulant was melt, which created a strain to delaminate the perovskite solar module at the interface of electron-transport layer (ETL) and metal electrode, such as epoxy resin as encapsulant in Fig. 2. The lead halide perovskite films and ETL stayed at the ITO/glass side, then ETL was washed by 1,2-dichlorobenzene (DCB), and lead halide perovskite was dissolved in DMF for subsequent lead recycling (Fig. 2b). After washing away the hole-transport layer and other residuals, ITO/glass could reuse for module refabrication. We found no noticeable conductivity changes for the ITO/glass substrate after recycling process. Even after annealing at 250 °C for 1 h, the conductivity of ITO/glass only slightly increased from 14.6 Ω/sq to 15.2 Ω/sq (Fig. 2c). The Cr and Cu electrodes with the encapsulant stayed at back cover-glass side, where 30 nm of Cr layer on the top surface induced a black color and electrode/encapsulant film formed winkles after thermal stress (Fig. 2b). The encapsulant and metal electrode were scraped by a knife when the encapsulant was still soft, and then the back cover glass was cleaned for reuse.

### Cation-exchange resins for lead recycling

**Fig. 3:**
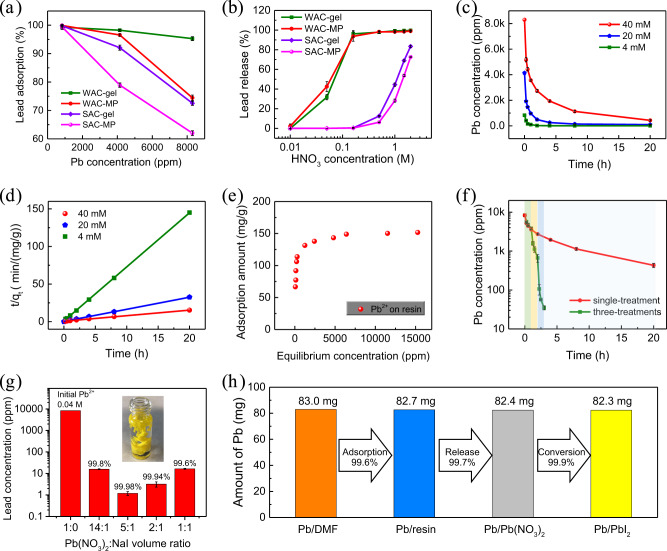
Lead recycling by cation-exchange resins. **a** Lead-adsorption ratio of 10 mL different concentrations of PbI_2_ solution in DMF after stirring with 1 g of cation-exchange resins for 20 h. **b** Lead-release ratio of 1 g of cation-exchange resins under different concentrations of HNO_3_ for 30 min. **c** Adsorption kinetics and (**d**) second-order kinetic fit for lead adsorption onto WAC-gel resin at different initial Pb concentrations. **e** The relation of adsorbed lead amount and equilibrium Pb^2+^ ion concentration in DMF solution, after adsorption for three days. **f** Lead adsorption for 10 mL of 40 mM lead in DMF by WAC-gel single treatment and three treatments. Single treatment was carried out with 1 g of WAC-gel for 20 h. Three treatments were carried out with 1 g of WAC-gel for 1 h, then the left PbI_2_ solution was transferred to 1 g of fresh resin for the second and third treatment. **g** Lead concentration in solution after mixing 0.04 M Pb(NO_3_)_2_ and 1.5 M NaI with different volume ratio. The inset is a photo of 1.5 M NaI solution dropped into Pb(NO_3_)_2_ containing solution. **h** Lead recycling ratio of 10 mL of 40 mM PbI_2_ in DMF by WAC-gel three treatments, lead release by HNO_3_ solution for 30 min, and lead conversion by reaction with NaI solution. The error bars in (**a**), (**b**), (**c**), (**f**), and (**g**) represent the standard deviation for three samples.

It is crucial to find a material with both high lead-adsorption efficiency from waste solution and high lead release ratio for the purpose of efficient lead recycling. We previously demonstrated that a cation-exchange resin layer integrated into the perovskite layer and onto the electrode can effectively avoid lead leakage when the modules are broken, where the strongly acidic cation-exchange resin with strong bonding between sulfonic acid and lead displayed excellent lead trapping effect^21,22^. We would like to start with the demonstrated lead adsorbent that can retain lead in perovskite modules during its operation lifetime. However, we find that the choice of lead absorber for lead cycling is different from previous studies for lead trapping, because it needs to release lead ions in this case where a lead absorber with appropriate bonding strength with lead may be preferred. Figure 3a–b compares the lead recycling performance using weakly acidic cation (WAC)-exchange resins with functional group of carboxylic acid based on both gel and macroporous (MP) matrix structures, and strongly acidic cation (SAC)-exchange resins with functional group of sulfonic acid based on gel and MP matrix. For 10 mL of 4 mM PbI_2_ (lead concentration of 830 parts per million (ppm)) in DMF, all four types of cation-exchange resins adsorbed more than 99.2% of Pb^2+^ ions from DMF solution after stirring with 1 g of resin for 20 h (Fig. 3a). When the initial lead concentration was increased to 40 mM, WAC-gel remained a high lead-adsorption ratio of 95%, while lead-adsorption ratio for other three cation-exchange resins dropped to less than 80% (Fig. 3a). For the lead-release process using HNO_3_ regenerant, Fig. 3b shows the carboxylic acid cation-exchange resins WAC-gel and WAC-MP both released most of adsorbed Pb^2+^ ions after 30 min of regeneration when the concentration of HNO_3_ regenerant is higher than 0.16 M. However, the sulfonic acid cation exchange resins SAC-gel and SAC-MP both showed a low lead-release ratio even when the concentration of HNO_3_ regenerant was up to 2 M (Fig. 3b).

The large total capacity of WAC resin and its high affinity for hydrogen ions are crucial to achieve both high lead-removal ratio during adsorption process and high lead-release ratio during regeneration process. The ion exchange between H^+^ and Pb^2+^ undergoing reversible reaction as shown in equation (1) and the equilibrium constant ${K}_{{\rm {H}}^{+}}^{{{{{{\rm{Pb}}}}}}^{2+}}$can be defined as:

2 $${K}_{{{{{{\rm{H}}}}}}^{+}}^{{{{{{\rm{Pb}}}}}}^{2+}}=\frac{{\left[{{{{{\rm{Pb}}}}}}^{2+}\right]}_{r}\times {\left[{{{{{\rm{H}}}}}}^{+}\right]}_{s}^{2}}{{\left[{{{{{\rm{Pb}}}}}}^{2+}\right]}_{s}\times {\left[{{{{{\rm{H}}}}}}^{+}\right]}_{r}^{2}}$$

where ${\left[{{{{{{\rm{Pb}}}}}}}^{2+}\right]}_{{{{{{\rm{r}}}}}}}$ and ${\left[{{{{{{\rm{H}}}}}}}^{+}\right]}_{{{{{{\rm{r}}}}}}}$ is the concentration of Pb^2+^ and H^+^ ions on resin, respectively, and ${\left[{{{{{{\rm{Pb}}}}}}}^{2+}\right]}_{{{{{{\rm{s}}}}}}}$ and ${\left[{{{{{{\rm{H}}}}}}}^{+}\right]}_{{{{{{\rm{s}}}}}}}$ is the concentration of Pb^2+^ and H^+^ ions in solution, respectively. The ratio of Pb^2+^ concentration on resin and Pb^2+^ concentration in solution is characterized with a distribution coefficient D:

3 $$D=\frac{{\left[{{{{{\rm{Pb}}}}}}^{2+}\right]}_{r}}{{\left[{{{{{\rm{Pb}}}}}}^{2+}\right]}_{s}}={K}_{{{{{{\rm{H}}}}}}^{+}}^{{{{{{\rm{Pb}}}}}}^{2+}}\times \frac{{\left[{{{{{\rm{H}}}}}}^{+}\right]}_{r}^{2}}{{\left[{{{{{\rm{H}}}}}}^{+}\right]}_{s}^{2}}$$

A large D value is needed for lead-adsorption process and a small D value is preferred during regeneration process, where the concentration difference of H^+^ on resin and in solution is also important besides ${K}_{{{{{{\rm{H}}}}}}^{+}}^{{{{{{\rm{Pb}}}}}}^{2+}}$ value. In lead-adsorption process, the large difference of H^+^ ion concentration on resin ${\left[{{{{{{\rm{H}}}}}}}^{+}\right]}_{{{{{{\rm{r}}}}}}}$ and in solution ${\left[{{{{{{\rm{H}}}}}}}^{+}\right]}_{{{{{{\rm{r}}}}}}}$ generates a large D value for efficient adsorption of Pb^2+^ ions. The WAC-gel and WAC-MP resin in this study has a high total capacity (total active H^+^ sites on resin) of 4 eq/L (equivalents per liter of resin), which is larger than the total capacity of 1.9 eq/L for SAC-gel resin and 1.7 eq/L for SAC-MP resin. This large ${\left[{{{{{{\rm{H}}}}}}}^{+}\right]}_{{{{{{\rm{r}}}}}}}$ on WAC resin explains its excellent lead-adsorption performance. During the resin-regeneration process, the high H^+^ ion concentration in HNO_3_ solution ${\left[{{{{{{\rm{H}}}}}}}^{+}\right]}_{{{{{{\rm{s}}}}}}}$ decreases the D value, thus releasing Pb^2+^ ions. The major difference between the WAC resin and SAC resin is their affinity of Pb^2+^ ions compared with H^+^ ions. Due to different acid-dissociation constant, the ${K}_{{{{{{\rm{H}}}}}}^{+}}^{{{{{{\rm{Pb}}}}}}^{2+}}$ for WAC resin based on carboxylic acid functional group is smaller than 1, while the ${K}_{{{{{{\rm{H}}}}}}^{+}}^{{{{{{\rm{Pb}}}}}}^{2+}}$ for SAC resin based on sulfonic acid functional group is larger than 1^42^. As a result, WAC resin can be regenerated to its full capacity with near 100% lead desorption by using a low concentration of HNO_3_ solution. However, the SAC resin is difficult to fully release the adsorbed Pb^2+^ ions even under high concentration of HNO_3_ solution. We thus chose the WAC resin for the lead recycling.

To better understand the lead-adsorption process on WAC-gel resin, the adsorption kinetics is characterized in Fig. 3c. During the rate-limiting step, if the adsorbed ion interacts with single unoccupied site on the adsorbent, the curve can be fitted with the pseudo-first-order kinetic model^43,44^:

4 $$\frac{d{q}_{t}}{dt}={k}_{1}\cdot ({q}_{e}-{q}_{t})$$

5 $$\log ({q}_{e}-{q}_{t})=\,\log ({q}_{e})-\left (\frac{{k}_{1}}{2.303}\right )t$$

where *q*_*e*_ and *q*_*t*_ the amount adsorbed at equilibrium and at time *t*, respectively (mg/g), and *k*_1_ is the rate constant (min^−1^). A pseudo-second-order kinetic model is based on sorption equilibrium that the adsorbed ion interacts with pairs of independent unoccupied sites on adsorbent during the rate-limiting step, and the kinetic rate is^43,44^:

6 $$\frac{d{q}_{t}}{dt}={k}_{2}\cdot {({q}_{e}-{q}_{t})}^{2}$$

7 $$\frac{t}{{q}_{t}}=\frac{1}{{k}_{2}{q}_{e}^{2}}+\frac{1}{{q}_{e}}t$$

where *k*_2_ is the rate constant (min^−1^). Figure 3d and Supplementary Fig. 1 show that the pseudo-second-order kinetic model is more matched than pseudo-first-order kinetic model for lead adsorption by WAC-gel resin. This indicates that the chemical adsorption of Pb^2+^ ions involves ion exchanges with two H^+^ sites on WAC-gel resin during the rate-limiting step.

In order to better characterize the adsorption performance, we analyzed the adsorption-operating capacity of WAC-gel resin under different Pb^2+^ ion concentrations. Different initial concentration of PbI_2_ solutions was prepared by dissolving 2 mmol of PbI_2_ powder in different amount of DMF solvent, then 1 g of WAC-gel resin was added into those PbI_2_ solutions under 400-rpm stir at room temperature for three days. Figure 3e shows the measured relation of adsorbed lead amount on WAC-gel resin and equilibrium Pb^2+^ ion concentration in solution. For 1 g of WAC-gel resin, it could adsorb more than 100 mg and around 150 mg of lead when the equilibrium Pb^2+^ concentration in solution is larger than 200 ppm and 6000 ppm, respectively.

The lead recycling speed and efficiency for high concentration PbI_2_ solution by carboxylic acid cation-exchange resin were analyzed. Because the lead-adsorption rate is determined by the number of active sites available on resin, the adsorption rate slows down when there are fewer active sites. In order to increase the lead-removal speed for high-concentration PbI_2_ solution, we utilized three treatments instead of single treatment with WAC-gel resin, where the left PbI_2_ solution was transferred to fresh resin for the second and third treatment. As a result, lead-removal ratio of 99.6% can be achieved for 40 mM lead solution after three WAC-gel treatments with one hour for each treatment (Fig. 3f). This greatly decreased the required time for efficient lead removal. It is noted that the lead-adsorption study in previous reported iron-incorporated hydroxyapatite as adsorbent was carried out at initial PbI_2_ concentration of 2 mM^37^, and the initial PbI_2_ concentration in this study was 20 times higher, which results in 20-times less-required solvents for the recycling process. This could greatly reduce the recycling cost, as will be discussed later that DMF is one of major recycling consumptions.

### Lead conversion from Pb(NO_3_)_2_ to PbI_2_

NaI solution was added into Pb(NO_3_)_2_ containing solution to convert the soluble Pb(NO_3_)_2_ to insoluble PbI_2_ as precipitation. Low solubility of PbI_2_ in aqueous solution, especially solution with additional I^-^ ions, is the key for high conversion ratio of lead from Pb(NO_3_)_2_ to PbI_2_. Supplementary Fig. 2 shows the measured lead concentration of PbI_2_ in water as a function of added iodide concentration. The Pb^2+^ ion concentration in saturated PbI_2_ water solution is 261.7 ppm. With the addition of NaI, the concentration of I^-^ ions in solution is increased, as a result, the concentration of Pb^2+^ ions in solution is decreased in order to maintain the solubility-product constant of K_sp_ = [Pb^2+^][I^−^]^2^. However, when the concentration of I^−^ ions is too high, it facilitates the formation of other types of soluble lead, such as ${{{{{{\rm{PbI}}}}}}}_{3}^{-}$ and ${{{{{{\rm{PbI}}}}}}}_{4}^{2-}$ (Supplementary Fig. 2b). For 0.04 M Pb(NO_3_)_2_ solution, Fig. 3g shows that after mixing the Pb(NO_3_)_2_ solution with 1.5 M NaI solution at volume ratio between 14:1 and 1:1 could convert more than 99.6% of Pb(NO_3_)_2_ to PbI_2_, and a volume ratio of 5:1 results in a reduced lead concentration to ~1 ppm, which corresponds to a theoretical maximum PbI_2_ precipitation yield of 99.98%. The yield of PbI_2_ precipitation from Pb(NO_3_)_2_ solution is calculated by the amount of Pb^2+^ ions in solution before and after adding NaI solution. Considering the lead-adsorption ratio of 99.6% and lead-release ratio of 99.7%, the overall lead-recycling ratio was 99.2% after lead adsorption, lead release, and lead conversion processes (Fig. 3h).

### Recycled materials’ properties

**Fig. 4:**
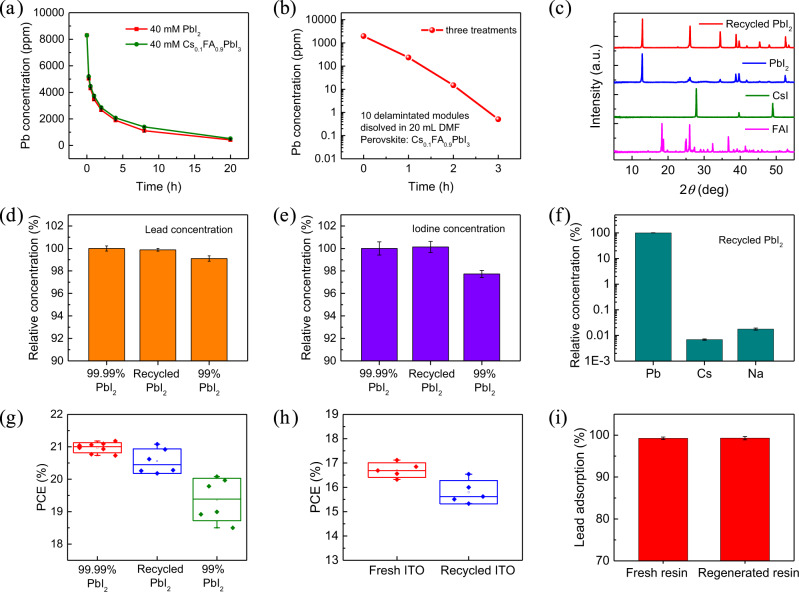
Recycling the perovskite solar module. **a** Lead-adsorption kinetics for 10 mL of 40 mM PbI_2_ and 40 mM Cs_0.1_FA_0.9_PbI_3_ solution by WAC-gel resin. **b** Lead adsorption for perovskite solution by three WAC-gel treatments with one hour at each treatment. The perovskite solution was prepared by dissolving 10 delaminated perovskite solar modules in 20 mL of DMF, and module active area was ~25.0 cm^2^. **c** XRD pattern of recycled PbI_2_ from Cs_0.1_FA_0.9_PbI_3_ solution, compared with XRD patterns of commercial PbI_2_, FAI, and CsI. **d** Relative lead concentration and (**e**) relative iodine concentration in DMF solution with the same amount of commercial 99.99% PbI_2_, recycled PbI_2_, and commercial 99% PbI_2_, where commercial 99.99% PbI_2_ was used as reference and concentration was measured by ICP-MS. **f** Ratios of Cs and Na to Pb for recycled PbI_2_ in DMF solution, measured by ICP-MS. **g** PCE of perovskite solar cells fabricated with commercial 99.99% PbI_2_, recycled PbI_2_, and commercial 99% PbI_2_, and device size was 8 mm^2^. **e** PCE of perovskite solar modules fabricated on fresh ITO/glass and recycled ITO/glass, and module active area was ~25.0 cm^2^. **f** Lead adsorption of regenerated WAC-gel compared with fresh WAC-gel. The error bars in (**a**), (**d**), (**e**), (**f**), and (**i**) represent the standard deviation for three samples. Box range in (**g**) and (**h**) is standard deviation and center line is median.

The lead-recycling performance of carboxylic acid cation-exchange resin for mixed-cation solution was investigated. It is important to selectively recycle PbI_2_ from the perovskite solution with mixed cations. We compared the lead adsorption speeds for PbI_2_ solution and mixed cation Cs_0.1_FA_0.9_PbI_3_ perovskite solution by WAC-gel in Fig. 4a, and they showed similar speed. This indicated that the Cs^+^ ions and FA^+^ ions did not reduce the lead-adsorption rate, which was attributed to the stronger bonding of Pb^2+^ ion to cation-exchange resin than other cations in perovskite solution. For 10 delaminated perovskite solar modules with composition of Cs_0.1_FA_0.9_PbI_3_ directly dissolved in 20 mL of DMF, the initial lead concentration was 1955 ppm, and it dropped to 0.5 ppm with a lead-adsorption ratio of 99.97% after three WAC-gel treatments. For mixed-cation Cs_0.1_FA_0.9_PbI_3_ perovskite solution, after ion exchanges for adsorption and desorption and subsequently reaction with NaI solution, the precipitation was pure PbI_2_ without CsI or FAI, as confirmed by XRD patterns (Fig. 4c). This is because CsI and FAI have large solubility in aqueous solution and do not form precipitation, even if Cs^+^ ions and FA^+^ ions were adsorbed and released by cation-exchange resins.

We compared the purity of recycled PbI_2_ powder with commercial PbI_2_ powders—99.99% purity from TCI and 99% purity from Sigma-Aldrich. For the recovered lead from perovskite photovoltaics in the format of PbI_2_, we denote it as recycled PbI_2_. For 184 mg of different types of PbI_2_ powder dissolved in 2 mL of DMF solvent, Fig. 4d and e shows that recycled PbI_2_ had 99.9% of relative lead concentration and 100% of relative iodine concentration compared with commercial 99.99% PbI_2_, and it had better purity than the commercial 99% PbI_2_. The ratios of Cs and Na to Pb for recycled PbI_2_ dissolved in DMF are both less than 0.1% (Fig. 4f). For the Cs_0.1_FA_0.9_PbI_3_ perovskite solar cells based on different type of PbI_2_, the median PCE was 21.0%, 20.4%, and 19.4% for 99.99% PbI_2_, recycled PbI_2_, and 99% PbI_2_, respectively (Fig. 4g). It shows that the purity of PbI_2_ powder influences the photovoltaic performance of perovskite solar cells, and the perovskite solar cells based on recycled PbI_2_ displayed comparable efficiency to the devices fabricated with commercial high-purity (99.99%) PbI_2_.

During the whole recycling processes, the lead, front ITO/glass, and back cover glass could be recycled from degraded perovskite solar modules. The perovskite solar cells based on recycled PbI_2_ and perovskite solar modules based on recycled ITO/glass displayed PCE close to devices fabricated on fresh commercial raw materials (Fig. 4g, h, and Supplementary Fig. 3). Supplementary Fig. 4 shows that the recycled PbI_2_ and recycled ITO/glass substrates did not compromise the photovoltaic stability of perovskite solar devices. Moreover, the DMF organic solvent and regenerated cation-exchange resin could be reused to reduce the cost of recycling process. The regenerated WAC-gel resin displayed similar lead-adsorption performance as the fresh resin (Fig. 4i). Supplementary Fig. 5 shows that the WAC-gel resin had excellent lead-adsorption performance in different organic solvents, aqueous solution, as well as solvents with a wide range of pH values. This makes it easy for the fresh and regenerated resin to recover lead from different types of lead-containing solutions.

### Impact of lead format on recycling

A small amount of PbO might form in end-of-life perovskite solar modules due to oxidization. Although PbO is not soluble in DMF, after washing the degraded perovskite film in DMF with ultrasonication, the PbO particles can be filtered and dissolved by HNO_3_ solution for recycling. Supplementary Fig. 6 shows that a lead-adsorption ratio of 99.9% by WAC-gel resin can be reached for the dissolved PbO in HNO_3_ solution with an initial lead concentration of 40 mM and pH value of 2.4. The excellent lead-adsorption performance of WAC-gel resin in acidic aqueous solution and organic solvent allows us to recycle lead from perovskite solar modules even when lead exists in different compounds, such as perovskites, PbI_2_, and PbO. Moreover, the majority of lead ions in end-of-life perovskite solar modules should still maintain as perovskite phase or PbI_2_. When perovskite photovoltaics are well encapsulated to prevent oxygen permeation, other degradation channels should dominate, such as the formation of defects, formation of non-perovskite phase, or decomposition of perovskite to PbI_2_. For the Cs_0.1_FA_0.9_PbI_3_ solar cells after illumination for 2000 hours whose PCE dropped to less than 80% of the initial PCE value, we did not find notable PbO formation, though the perovskite film already changed to yellow color due to formation of δ-phase (Supplementary Fig. 7).

### Cost analysis

A technoeconomic assessment was carried out to understand the potential cost saving for the recycling technology for perovskite solar modules. Here we mainly consider the material cost. As shown in Table 1, the calculated total material cost for perovskite solar module based on structure in Fig. 2a was ~$24.8 /m^2^, similar as the cost modeling by Li et al. and Cai et al.^45,46^. The total value of recycled components, including front ITO/glass, PbI_2_, and back cover glass, was around $12/m^2^. Since the perovskite raw material itself constitutes a very small portion of the material cost in perovskite solar modules, the saving of material cost from recycled lead as PbI_2_ is not large. The majority of the material cost saving comes from expensive ITO glass and cover glass^45–51^. The recycling process does consume materials, including DMF, cation-exchange resin, DCB, HNO_3_, and NaI, while some of them can be reused for multiple cycles. For 1-m^2^ perovskite solar module with 1-μm-thick lead halide perovskite layer, it needs around 63 g of DMF to dissolve them for 0.1 M perovskite solution, 20 g of resin for three WAC-gel-adsorption treatments, 4 g of DCB for C_60_ removal^52^, 2.5 g of nitric acid for lead release, and 2.7 g of NaI for lead conversion. Based on this recycling process, the material consumption for recycling the perovskite solar module was $4.24 /m^2^ if these materials were used only once. The material cost could further be decreased to $1.35 /m^2^ if reusing DMF and resin for five times, which is around one order of magnitude lower than the overall value of recycled components. Besides removal of the toxic lead from end-of-life perovskite solar modules to avoid the environmental pollution, this recycling technique brings in dramatic revenue to make recycling compelling. Recycled components could provide energy savings compared with the production of new materials, and they provide another source of raw materials that is not dependent on primary mining and could relieve some supply chain constraints as well.

**Table 1:** Cost estimation.

Module materials	Price ($/kg)	Weight (g/m^2^)	Cost($/m^2^)	Reference
ITO/glass (0.67–3.2 mm)	1.28 (1.0–5.0)	5000 (1675–8000)	6.4 (6.4–12)	^45–51^
PTAA (8 nm)	352500	0.015	5.29	^(a)^
PbI_2_ (Perovskite 1 µm)	1028	3.12	3.21	^(b)^
FAI (Perovskite 1 µm)	1480	1.05	1.55	^(c)^
CsI (Perovskite 1 µm)	2080	0.18	0.37	^(b)^
C_60_ (30 nm)	59950	0.05	3.00	^(a)^
BCP (6 nm)	72200	0.007	0.51	^(d)^
Cr (30 nm)	274	0.22	0.06	^(e)^
Cu (150 nm)	252	1.34	0.34	^(e)^
Encapsulant (150–400 µm)	5.3 (4.6–14.2)	320 (138-400)	1.7 (1.54–2.0)	^45–51^
Back glass (2–2.5 mm)	0.48 (0.4–1.0)	5000 (5000–6250)	2.4 (2.4–5.04)	^45–51^
Total materials			24.8	

Recycled components	Price ($/kg)	Weight (g/m^2^)	Cost($/m^2^)	Reference
ITO/glass (0.67–3.2 mm)	1.28 (1.0–5.0)	5000 (1675–8000)	6.4 (6.4–12)	^45–51^
PbI_2_	1028	3.12	3.21	^(b)^
Back glass (2–2.5 mm)	0.48 (0.4–1.0)	5000 (5000–6250)	2.4 (2.4–5.04)	^45–51^
Total recycled			12.0	

Recycling consumption	Price ($/kg)	Weight (g/m^2^)	Cost($/m^2^)	Reference
DMF (reusable)	38	63	2.41	^(b)^
Resin (reusable)	60	20	1.20	^(f)^
DCB	22	4	0.09	^(b)^
HNO_3_	18.6	2.5	0.05	^(b)^
NaI	183	2.7	0.49	^(b)^
Total consumption			4.24	
Total consumption (reuse DMF and resin for 5 times)			1.35	

Note: Sale price from (a) Solaris Chem, (b) Sigma-Aldrich, (c) Greatcell Solar, (d) TCI America, (e) Kurt J. Lesker Company, and (f) ResinTech Inc.

Material cost for perovskite solar modules based on 1 µm Cs_0.1_FA_0.9_PbI_3_ film with device structure as shown in Fig. 2a; recover value of recycled components from perovskite solar modules; and material consumption cost for the proposed recycling process.

## Discussion

In conclusion, we developed a recycling technology for end-of-life perovskite solar modules that not only recycled the toxic lead to avoid the environmental pollution but also recycled valuable glass components as cost-effective method. The recycling process included thermal delamination to disassemble modules with intact glass substrates and efficient ion exchange to separate and recycle lead from organic solvent. The carboxylic acid cation-exchange resin showed high lead-adsorption ratio to separate lead from lead-containing solution, as well as high lead-release ratio during resin-regeneration process to recover lead ions as soluble Pb(NO_3_)_2_ and then convert to PbI_2_ precipitate for reuse. This method could recycle both the toxic lead and valuable ITO/glass and back cover-glass substrates from decommissioned perovskite solar modules for device refabrication. There was no obvious photovoltaic performance drop for the perovskite solar devices based on recycled PbI_2_ or recycled ITO/glass compared with the fresh counterparts. This provides a cost-effective recycling method for a close-loop lead management for perovskite solar modules to avoid the environmental pollution, which could significantly accelerate the penetration of perovskite photovoltaic technologies into the clean and renewable energy market.

## Methods

### Materials

Carboxylic acid cation-exchange resin WAC-gel (WACG-HP, gel type, hydrogen form) and WAC-MP (WACMP, macroporous type, hydrogen form) and sulfonic acid cation-exchange resin SAC-MP (SACMP-H, macroporous type, hydrogen form) were purchased from ResinTech Inc. Sulfonic acid cation-exchange resin SAC-gel (Amberlite^®^ IRC120 H, gel type, hydrogen form), Lead(II) iodide (99%), PTAA (average Mn 7000–10,000), bathocuproine (BCP), dimethylformamide (DMF), dimethyl sulfoxide (DMSO), 2-methoxyethanol (2-ME), 1,2-Dichlorobenzene, toluene, isopropyl alcohol, phenylethyl ammonium chloride, and Pb standard solution (1000 ± 2 ppm) were purchased from Sigma-Aldrich. Lead(II) iodide (99.99%, trace-metal basis) was purchased from TCI America. Formamidinium iodide (FAI) and formamidinium chloride were purchased from GreatCell Solar. C_60_ was purchased from Nano-C Inc. Copper (Cu) and chromium (Cr) for thermal evaporation were purchased from Kurt J. Lesker company.

### Fabrication of perovskite solar cells and modules

Prepatterned ITO glass substrates (1.5 cm by 1.5 cm for solar cells and 6.5 cm by 8.5 cm for solar modules) were first cleaned by ultrasonication with soap, deionized water, isopropyl alcohol, and acetone, and then UV ozone was treated for 15 min before use. The PTAA solution with a concentration of 2.2 mg/mL in toluene was blade-coated on ITO/glass substrate at 20 mm/s with 200-µm coating gap. The perovskite film coating was similar to previous studies^9^. The Cs_0.1_FA_0.9_PbI_3_ perovskite films were blade-coated at 20 mm/s with 250-µm coating gap at room temperature under nitrogen knife blowing by using a precursor solution containing 1.0 M FAPbI_3_ and 0.11 M CsPbI_3_ dissolved in a 2-ME/DMSO solvent mixture, where the precursor was filtered by PTFE filter with pore size of 0.2 µm before use. Formamidinium hypophosphite, formamidinium chloride, and phenylethyl ammonium chloride were added into the solution as additives at molar percentages of ~1.0%, ~1.5%, and ~0.15% to Pb^2+^ ions, respectively. The as-coated solid film was annealed at 150 °C in air for 3 min. Then the perovskite films were thermally evaporated with C_60_ (30 nm), BCP (6 nm), and Cu (150 nm) to complete the perovskite solar cell fabrication. For fabricating modules, laser ablation was applied before and after the deposition of the metal electrode (30 nm of Cr and 150 nm of Cu) to complete the series interconnection between subcells in the module.

### Module encapsulation and delamination

Perovskite solar module was encapsulated by a back cover glass using a Gorilla epoxy coated at the top sides of the glass and then cured for one night. The encapsulant layer thickness after drying was around 300 µm. The module delamination was carried out by placing the encapsulated perovskite solar module on a hot plate at 250 °C for 2 min. The encapsulant was softened and melt, and then a knife blade inserted between ITO/glass substrate and back cover glass at one corner of the module to detach the two glass substrates. The perovskite layer on delaminated ITO/glass substrate was dissolved by DMF for the subsequently lead-recycling process.

### Lead-recycling test

Different lead sources were used for lead-adsorption measurement: PbI_2_, Cs_0.1_FA_0.9_PbI_3_, Pb(NO_3_)_2_, and delaminated perovskite solar modules with Cs_0.1_FA_0.9_PbI_3_ perovskite film. Lead-adsorption ratios of different cation-exchange resins were characterized through measuring lead concentration change of 10 mL of 4–40 mM PbI_2_ solution in DMF after stirring with 1 g of cation-exchange resin under 400 rpm for different times. For the WAC-gel three treatments, 10 mL of lead-containing solution was stirred with 1 g of WAC-gel under 400 rpm for one hour, then transfer the left lead-containing solution to the second 1 g of fresh WAC-gel resin for the second hour and again to the third 1 g of fresh resin for the third hour. To characterize the resin adsorption capacity for Pb^2+^ ions in DMF solution, the adsorption processes were carried out by mixing 2 mmol of PbI_2_ powder in different amount of DMF solvent with 1 g of WAC-gel resin under stirring for three days. Lead-release process was carried out by stirring lead-adsorbed resin with 10 mL of different concentrations of aqueous HNO_3_ at 400 rpm for 30 min. After lead release, the regeneration solution together with the released Pb ions was transferred to precipitation bath, where 1.5 M NaI aqueous solution was added into it and formed yellow PbI_2_ precipitate. The PbI_2_ precipitate was washed by deionized water and isopropyl alcohol and collected by centrifugation, and then dried under vacuum before reuse. The regenerated WAC-gel was rinsed by deionized water before reuse for lead adsorption. The Pb and other elements concentration in solution was measured by inductively coupled plasma mass spectrometry (ICP-MS) Nexion 300D instrument. Before the measurement, the calibration curve for analysis with concentration between 1 part per billion (ppb) and 100 ppb was drawn by measuring standard solutions prepared by mixing the standard solution with different amount of 2% HNO_3_ aqueous solution. For each measurement, the Pb concentration of the tested solution was diluted by 2% HNO_3_ aqueous solution to 1 ppb and 100 ppb within the linear calibration curve of ICP-MS.

### Device characterizations

The power conversion efficiency of perovskite solar cell and solar module was characterized by J–V measurement under a xenon lamp based solar simulator (Oriel Sol3A, Class AAA Solar Simulator). The light intensity was calibrated to 100 mW cm^−2^ by a silicon reference cell (Newport 91150-KG5). For perovskite solar cells, a metal mask with an aperture area of 6.08 mm^2^ (3.8 mm × 1.6 mm) aligned with the device area. The J–V curves were measured by a Keithley 2400 source meter with a backward and forward scan rate of 0.1 V s^−1^ with a delay time of 10 ms in air at room temperature. To measure the long-term operational stability, the encapsulated devices and modules was illuminated by a 1-sun-equivalent LED light. External quantum efficiency data were obtained with a Newport QE measurement kit by focusing a monochromatic beam of light onto the device. The XRD patterns were obtained by a Rigaku sixth-generation MiniFlex X-ray diffractometer.

### Reporting summary

Further information on experimental design is available in the Nature Research Reporting Summary linked to this paper.

## Supplementary information

Supplementary Information

Solar Cells Reporting Summary

## Data Availability

The data that support the findings of this study are available from the corresponding author upon request.

## References

[1] Jena AK, Kulkarni A, Miyasaka T (2019). Halide perovskite photovoltaics: Background, status, and future prospects. Chem. Rev..

[2] Huang J, Yuan Y, Shao Y, Yan Y (2017). Understanding the physical properties of hybrid perovskites for photovoltaic applications. Nat. Rev. Mater..

[3] NREL. https://www.nrel.gov/pv/cell-efficiency.html. (2021).

[4] Deng Y (2019). Tailoring solvent coordination for high-speed, room-temperature blading of perovskite photovoltaic films. Sci. Adv..

[5] Subbiah AS (2020). High-performance perovskite single-junction and textured perovskite/silicon tandem solar cells via slot-die-coating. ACS Energy Lett..

[6] Das S (2015). High-performance flexible perovskite solar cells by using a combination of ultrasonic spray-coating and low thermal budget photonic curing. ACS Photonics.

[7] Yin W-J, Shi T, Yan Y (2014). Unusual defect physics in CH_3_NH_3_PbI_3_ perovskite solar cell absorber. Appl. Phys. Lett..

[8] Li Z (2018). Scalable fabrication of perovskite solar cells. Nat. Rev. Mater..

[9] Deng Y (2021). Defect compensation in formamidinium-cesium perovskites for highly efficient and stable solar modules. Nat. Energy.

[10] Extance A (2019). The reality behind solar power’s next star material. Nature.

[11] Lu HZ (2020). Vapor-assisted deposition of highly efficient, stable black-phase FAPbI_3_ perovskite solar cells. Science.

[12] Jeong J (2021). Pseudo-halide anion engineering for alpha-FAPbI_3_ perovskite solar cells. Nature.

[13] Jung EH (2019). Efficient, stable and scalable perovskite solar cells using poly(3-hexylthiophene). Nature.

[14] Jiang Q (2019). Surface passivation of perovskite film for efficient solar cells. Nat. Photonics.

[15] Chen SS, Xiao X, Gu HY, Huang JS (2021). Iodine reduction for reproducible and high-performance perovskite solar cells and modules. Sci. Adv..

[16] Hao F, Stoumpos CC, Cao DH, Chang RPH, Kanatzidis MG (2014). Lead-free solid-state organic-inorganic halide perovskite solar cells. Nat. Photonics.

[17] Jiang XY (2020). Ultra-high open-circuit voltage of tin perovskite solar cells via an electron transporting layer design. Nat. Commun..

[18] Yang XQ, Wang W, Ran R, Zhou W, Shao ZP (2020). Recent advances in Cs_2_AgBiBr_6_-based halide double perovskites as lead-free and inorganic light absorbers for perovskite solar cells. Energy Fuels.

[19] IRENA. https://irena.org/-/media/Files/IRENA/Agency/Publication/2019/Apr/IRENA_Global_Energy_Transformation_2019.pdf. (2019).

[20] Li X (2020). On-device lead sequestration for perovskite solar cells. Nature.

[21] Chen S (2020). Trapping lead in perovskite solar modules with abundant and low-cost cation-exchange resins. Nat. Energy.

[22] Chen S (2021). Preventing lead leakage with built-in resin layers for sustainable perovskite solar cells. Nat. Sustain..

[23] Heath GA (2020). Research and development priorities for silicon photovoltaic module recycling to support a circular economy. Nat. Energy.

[24] Deng R, Chang NL, Ouyang Z, Chong CM (2019). A techno-economic review of silicon photovoltaic module recycling. Renew. Sustain. Energy Rev..

[25] Chowdhury MS (2020). An overview of solar photovoltaic panels’ end-of-life material recycling. Energy Strategy Rev..

[26] Liu F-W (2021). Recycling and recovery of perovskite solar cells. Mater. Today.

[27] Zhang S (2018). Cyclic utilization of lead in carbon-based perovskite solar cells. ACS Sustain. Chem. Eng..

[28] Kim BJ (2016). Selective dissolution of halide perovskites as a step towards recycling solar cells. Nat. Commun..

[29] Poll CG (2016). Electrochemical recycling of lead from hybrid organic–inorganic perovskites using deep eutectic solvents. Green. Chem..

[30] Kadro JM (2016). Proof-of-concept for facile perovskite solar cell recycling. Energy Environ. Sci..

[31] Gonzalez M, Trócoli R, Pavlovic I, Barriga C, La Mantia F (2016). Capturing Cd (II) and Pb (II) from contaminated water sources by electro-deposition on hydrotalcite-like compounds. Phys. Chem. Chem. Phys..

[32] Kim T-y., Jeung S-Y, Cho S-Y, Kang Y, Kim S-J (2004). Removal and regeneration of heavy metal ions using cation exchange column. J. Ind. Eng. Chem..

[33] Goel J, Kadirvelu K, Rajagopal C, Garg VK (2005). Removal of lead (II) by adsorption using treated granular activated carbon: batch and column studies. J. Hazard. Mater..

[34] Matlock MM, Howerton BS, Atwood DA (2002). Chemical precipitation of lead from lead battery recycling plant wastewater. Ind. Eng. Chem. Res..

[35] Dean JG, Bosqui FL, Lanouette KH (1972). Removing heavy metals from waste water. Environ. Sci. Technol..

[36] Abdullah N (2016). Polysulfone/hydrous ferric oxide ultrafiltration mixed matrix membrane: preparation, characterization and its adsorptive removal of lead (II) from aqueous solution. Chem. Eng. J..

[37] Park SY (2020). Sustainable lead management in halide perovskite solar cells. Nat. Sustain..

[38] Deng Y (2020). Reduced self-doping of perovskites induced by short annealing for efficient solar modules. Joule.

[39] Shi L (2020). Gas chromatography-mass spectrometry analyses of encapsulated stable perovskite solar cells. Science.

[40] Cheacharoen R (2018). Encapsulating perovskite solar cells to withstand damp heat and thermal cycling. Sustain. Energy Fuels.

[41] Jiang Y (2019). Reduction of lead leakage from damaged lead halide perovskite solar modules using self-healing polymer-based encapsulation. Nat. Energy.

[42] Kunin R, Barry RE (1949). Carboxylic, weak acid type, cation exchange resin. Ind. Eng. Chem..

[43] Fil BA, Boncukcuoğlu R, Yilmaz AE, Bayar S (2012). Adsorption of Ni (II) on ion exchange resin: Kinetics, equilibrium and thermodynamic studies. *Korean*. J. Chem. Eng..

[44] Rengaraj S, Kim Y, Joo CK, Choi K, Yi J (2004). Batch adsorptive removal of copper ions in aqueous solutions by ion exchange resins: 1200H and IRN97H. *Korean*. J. Chem. Eng..

[45] Li Z (2018). Cost analysis of perovskite tandem photovoltaics. Joule.

[46] Cai M (2016). Cost-performance analysis of perovskite solar modules. Adv. Sci..

[47] Chang NL (2018). Manufacturing cost and market potential analysis of demonstrated roll-to-roll perovskite photovoltaic cell processes. Sol. Energy Mater. Sol. Cells.

[48] Chang NL (2017). A manufacturing cost estimation method with uncertainty analysis and its application to perovskite on glass photovoltaic modules. Prog. Photovolt..

[49] Song ZN (2017). A technoeconomic analysis of perovskite solar module manufacturing with low-cost materials and techniques. Energy Environ. Sci..

[50] Horowitz, K. A. et al. An analysis of the cost and performance of photovoltaic systems as a function of module area (National Renewable Energy Lab, 2017).

[51] Louwen A, Van Sark W, Schropp R, Faaij A (2016). A cost roadmap for silicon heterojunction solar cells. Sol. Energy Mater. Sol. Cells.

[52] Wang CI, Hua CC (2015). Solubility of C_60_ and PCBM in organic solvents. J. Phys. Chem. B.

