# Supplementary material for: Recycling lead and transparent conductors from perovskite solar modules
Source: Nat Commun. 2021 Oct 6;12:5859. doi: 10.1038/s41467-021-26121-1 (PMC8494795; doi:10.1038/s41467-021-26121-1)
Supplement: Supplementary file 1 — Supplementary Information [file 41467_2021_26121_MOESM1_ESM.pdf]

Supplementary Information for

Recycling Lead and Transparent Conductors from Perovskite Solar Modules

Bo Chen[1], Chengbin Fei[1], Shangshang Chen[1], Hangyu Gu[1], Xun Xiao[1], Jinsong Huang[1,*]

[1] Department of Applied Physical Sciences, University of North Carolina at Chapel Hill, Chapel Hill, NC 27599, USA

[*] Correspondence to J.H. (email: jhuang@unc.edu)

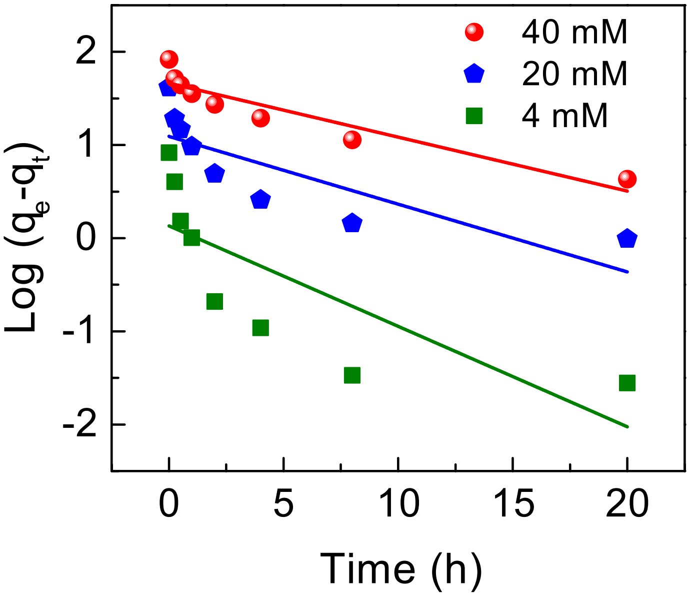

Supplementary Figure 1. *Pseudo-first order kinetic fit for lead adsorption by WAC-gel resin at different initial Pb concentrations*.

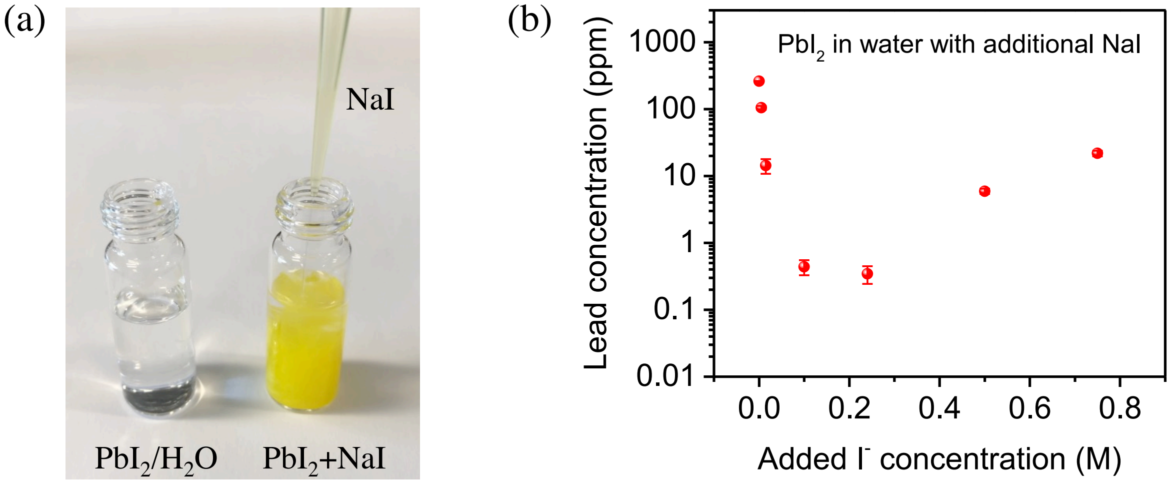

Supplementary Figure 2. *(a) Left: 3 mL PbI_2 water solution, right: 3 mL PbI_2 water solution with additional 0.2 mL of 1.5 M NaI solution. (b) Total soluble lead concentration in solution as a function of added iodide concentration with NaI, and lead concentration in solution was measured by ICP-MS. The error bars represent the standard deviation for three samples.*

There are different types of soluble lead in solution due to the following equations and solubility products:

$$PbI_2\,(s) \overset{K_{sp}}{\longleftrightarrow} Pb^{2+} + 2I^- \qquad K_{sp} = [Pb^{2+}][I^-]^2$$

$$Pb^{2+} + I^- \overset{K_1}{\leftrightarrow} PbI^+ \qquad K_1 = [PbI^+]/[Pb^{2+}][I^-]$$

$$Pb^{2+} + 2I^- \overset{\beta_2}{\leftrightarrow} PbI_2(aq) \qquad \beta_2 = [PbI_2(aq)]/[Pb^{2+}][I^-]^2$$

$$Pb^{2+} + 3I^- \overset{\beta_3}{\leftrightarrow} PbI_3^- \qquad \beta_3 = [PbI_3^-]/[Pb^{2+}][I^-]^3$$

$$Pb^{2+} + 4I^- \overset{\beta_4}{\leftrightarrow} PbI_4^{2-} \qquad \beta_4 = [PbI_4^{2-}]/[Pb^{2+}][I^-]^4$$

Therefore, the concentration of Pb^{2+} and PbI^+ in solution decreases with the increase of concentration of I^- ions, while the concentration of PbI_3^- and PbI_4^{2-} in solution increases with the increase of concentration of I^- ions, with the following relations:

$$[Pb^{2+}] = K_{sp}/[I^-]^2$$

$$[PbI^+] = K_1 K_{sp}/[I^-]$$

$$[PbI_2(aq)] = \beta_2 K_{sp}$$

$$[PbI_3^-] = \beta_3[Pb^{2+}][I^-]^3 = \beta_3 K_{sp}[I^-]$$

$$[PbI_4^{2-}] = \beta_4[Pb^{2+}][I^-]^4 = \beta_4 K_{sp}[I^-]^2$$

Supplementary Figure 2a shows that add NaI solution in PbI_2 water solution caused the precipitation of yellow PbI_2, due to the solubility of PbI_2 was reduced with present of additional iodide. Supplementary Figure 2b shows the measured total soluble lead concentration in solution by ICP-MS as function of added iodide concentration. When the concentration of added NaI was 0.1-0.25 M, solubility of PbI_2 in aqueous solution was lowest, and the lead concentration in water was significantly reduced from 261.7 ppm to less than 1 ppm. When the concentration of added I^- ions was higher than 0.4 M, it facilitated the formation PbI_3^- and PbI_4^{2-}, thus increased the concentration of total soluble lead in water as shown in Supplementary Figure 2b.

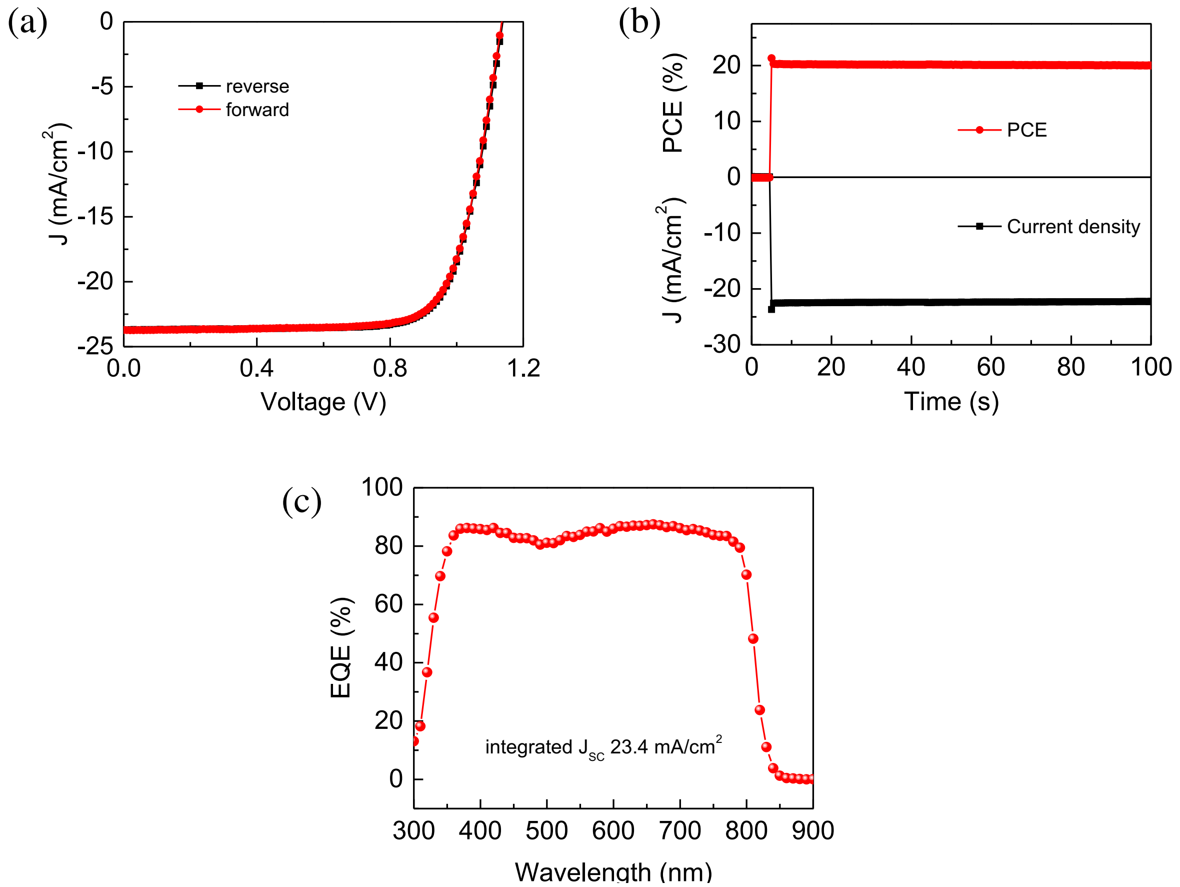

Supplementary Figure 3. *(a) J-V curves of perovskite solar cells based on recycled* PbI_2 *under reverse scan and forward scan. (b) Measured current density and PCE at the maximum power point with 0.9 V bias for perovskite solar cells based on recycled* PbI_2*. (c) EQE curve and integrated* J_{SC} *for perovskite solar cells based on recycled* PbI_2*.*

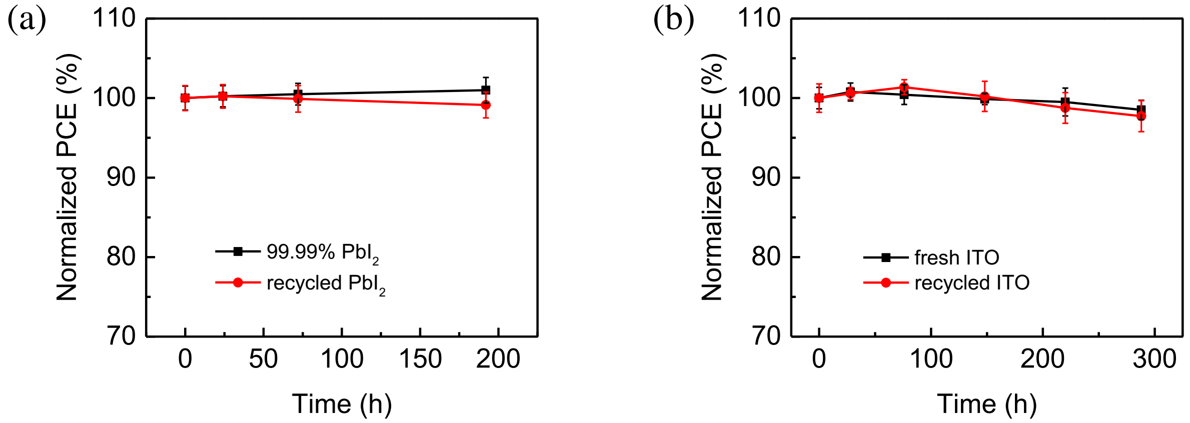

Supplementary Figure 4. *(a) Stability of perovskite solar cells based on commercial 99.99%* PbI_2 *and recycled* PbI_2*. (b) Stability of perovskite solar modules based on fresh ITO/glass and recycled ITO/glass. The error bars represent the standard deviation for three samples.*

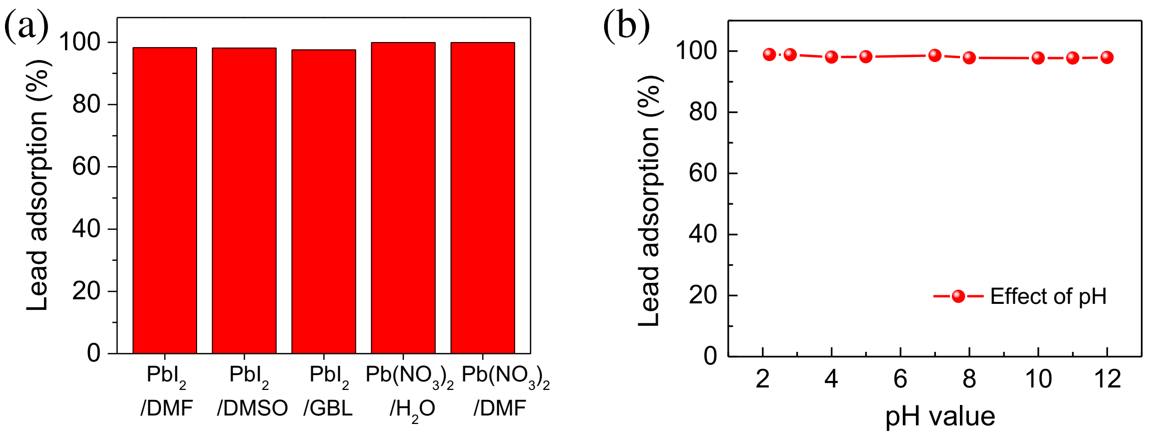

Supplementary Figure 5. *Lead adsorption ratio of 20 mM lead containing solution (a) at different organic solvents and water and (b) at solvent of DMF:water=9:1 with different pH values. The lead adsorption treatment was carried out by stirring 10 mL 20 mM of lead containing solution with 1 g WAC-gel resin under 400 rpm for 20 hours.*

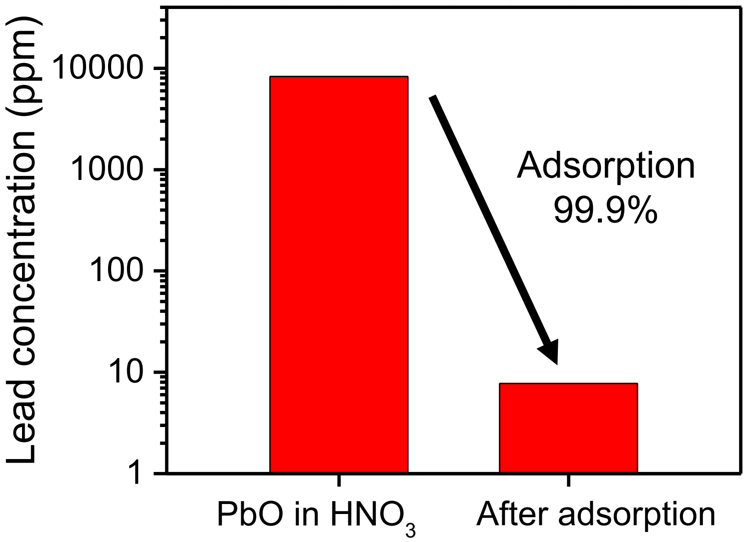

Supplementary Figure 6. *Lead adsorption performance of WAC-gel resin for PbO in* HNO_3 *solution. The initial solution with lead concentration of 40 mM and pH value of 2.4 was prepared by dissolving PbO powder in aqueous* HNO_3 *solution. The lead adsorption process was carried out with 1 g WAC-gel resin for 10 mL solution with stirring for 20 hours.*

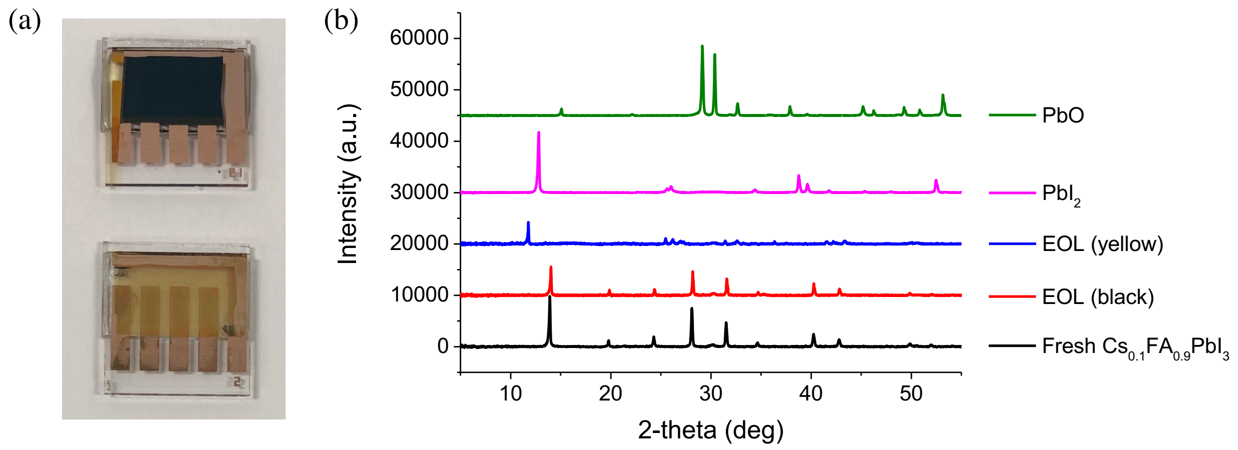

Supplementary Figure 7. *(a) Photo of end-of-life (EOL) $Cs_{0.1}FA_{0.9}PbI_3$ perovskite solar cells after 2000 hours of illumination with active layer in black color (top) and yellow color (bottom). (b) XRD patterns for fresh, EOL device with black active layer (EOL (black)), EOL device with yellow active layer of $Cs_{0.1}FA_{0.9}PbI_3$ perovskite solar cells (EOL (yellow)), commercial PbI_2 powder, and commercial PbO powder. The XRD peaks in EOL (yellow) are assigned to yellow δ-phase.*
