# Supplementary material for: Recycling lead and transparent conductors from perovskite solar modules
Source: Nat Commun. 2021 Oct 6;12:5859. doi: 10.1038/s41467-021-26121-1 (PMC8494795; doi:10.1038/s41467-021-26121-1)
Supplement: Supplementary file 2 — Solar Cells Reporting Summary [file 41467_2021_26121_MOESM2_ESM.pdf]

nature research

Corresponding author(s): Jinsong Huang

Solar Cells Reporting Summary

Nature Research wishes to improve the reproducibility of the work that we publish. This form is intended for publication with all accepted papers reporting the characterization of photovoltaic devices and provides structure for consistency and transparency in reporting. Some list items might not apply to an individual manuscript, but all fields must be completed for clarity.

For further information on Nature Research policies, including our data availability policy, see Authors & Referees.

Experimental design

Please check: are the following details reported in the manuscript?

1. Dimensions

Item	Yes	No	Details
Area of the tested solar cells	☒	☐	8mm2 for solar cells and 25.0 cm2 for modules.
Method used to determine the device area	☒	☐	Device characterizations part in Methods.

2. Current-voltage characterization

Item	Yes	No	Details
Current density-voltage (J-V) plots in both forward and backward direction	☒	☐	Supplementary Figure 3.
Voltage scan conditions<br>*For instance: scan direction, speed, dwell times*	☒	☐	Device characterizations part in Methods.
Test environment<br>*For instance: characterization temperature, in air or in glove box*	☒	☐	Device characterizations part in Methods.
Protocol for preconditioning of the device before its characterization	☒	☐	Device characterizations part in Methods.
Stability of the J-V characteristic<br>*Verified with time evolution of the maximum power point or with the photocurrent at maximum power point; see ref. 7 for details.*	☒	☐	Supplementary Figure 3.

3. Hysteresis or any other unusual behaviour

Item	Yes	No	Details
Description of the unusual behaviour observed during the characterization	☐	☒	No unusual behaviour was observed.
Related experimental data	☒	☐	Supplementary Figure 3.

4. Efficiency

Item	Yes	No	Details
External quantum efficiency (EQE) or incident photons to current efficiency (IPCE)	☒	☐	Supplementary Figure 3.
A comparison between the integrated response under the standard reference spectrum and the response measure under the simulator	☒	☐	Supplementary Figure 3.
For tandem solar cells, the bias illumination and bias voltage used for each subcell	☐	☒	No tandem solar cells.

5. Calibration

Item	Yes	No	Details
Light source and reference cell or sensor used for the characterization	☒	☐	Device characterizations part in Methods.
Confirmation that the reference cell was calibrated and certified	☒	☐	Device characterizations part in Methods.

Calculation of spectral mismatch between the reference cell and the devices under test

☐ Yes
☒ No

We mainly focus on the recycling, we have not done the spectral mismatch, which could result in small difference in photocurrent.

6. Mask/aperture

Size of the mask/aperture used during testing

☒ Yes
☐ No

An aperture of 6.08 mm2 was used during testing perovskite solar cells.

Variation of the measured short-circuit current density with the mask/aperture area

☐ Yes
☒ No

The aperture area is fixed for each device.

7. Performance certification

Identity of the independent certification laboratory that confirmed the photovoltaic performance

☐ Yes
☒ No

We mainly focus on the material recycling, and we did not aim to claiming record efficiency.

A copy of any certificate(s)
Provide in Supplementary Information

☐ Yes
☒ No

We mainly focus on the material recycling, and we did not aim to claiming record efficiency.

8. Statistics

Number of solar cells tested

☒ Yes
☐ No

Figure 4g and 4h.

Statistical analysis of the device performance

☒ Yes
☐ No

Figure 4g and 4h.

9. Long-term stability analysis

Type of analysis, bias conditions and environmental conditions
For instance: illumination type, temperature, atmosphere humidity, encapsulation method, preconditioning temperature

☒ Yes
☐ No

Supplementary Figure 4.
